# Augmentation of Psychotherapy through Alternative Preconscious Priming: A Case Series Exploring Effects on Residual Symptoms

**DOI:** 10.3389/fpsyt.2017.00008

**Published:** 2017-01-31

**Authors:** Melha Zidani, Jean-Sébastien Audet, François Borgeat, Frederick Aardema, Kieron Philip O’Connor, Yasser Khazaal

**Affiliations:** ^1^Department of Psychiatry, University of Montreal, Montreal, Canada; ^2^Research Center of the Montreal University Institute of Mental Health, Montreal, Canada; ^3^Montreal University Institute of Mental Health, Montreal, Canada; ^4^University of Geneva, Geneva, Switzerland; ^5^Geneva University Hospitals, Geneva, Switzerland

**Keywords:** priming, preconcious, social anxiety disorder/social phobia, generalized anxiety disorder, psychotherapy research

## Abstract

The current paper describes a case series using a new strategy for facilitating change based on Augmentation of Psychotherapy through Alternative Preconscious Priming (APAP) (1) in the treatment of eight treatment-resistant patients suffering from social phobia or generalized anxiety disorder. The patients had previously only shown a partial response to cognitive behavioral therapy (CBT) despite good treatment adherence. The patients completed APAP using a computerized program, which consisted of three steps during which alternative, more functional thoughts and beliefs relevant to the idiosyncratic difficulties experienced by the patients were formulated. Subsequently, these formulations were recorded and mixed with masking relaxing music, which the patient listened to in a passive state twice daily for 20 min for a period of 8 weeks. This case series aimed to assess the effect and acceptability of APAP using quantitative and qualitative measures administered before, after, and 16 weeks’ posttreatment. Results showed a reduction in dysfunctional idiosyncratic thoughts reported by most patients, as well as mild improvements in anxiety and important improvements in quality of life. APAP could be a valuable addition to CBT by facilitating or enhancing cognitive and symptom change. Further studies are needed to confirm these promising results.

## Introduction

Anxiety disorders, as defined by the fifth edition of the Diagnostic and Statistical Manual of the American Psychiatric Association (2), represent the most frequent mental health problems (3). According to the public health agency of Canada, anxiety disorders currently affect about 12% of the population and create light to severe impairment (4). Their lifetime prevalence in the general population varies between 16 and 29% (5). Anxiety disorders also represent a major economic burden for society in terms of direct costs, such as psychiatric or general health services, and indirect costs, such as lost of productivity and unemployment (6).

### Treatment of Anxiety Disorders

Treatment for anxiety disorder usually consists of antidepressant and cognitive behavioral therapy (CBT) (7). CBT stipulates that cognitions are modifiable through reasoning and that these modifications produce change in emotions and behaviors, which are central in psychiatric disorders (8). CBT is the most studied form of psychotherapy and seems to be the most effective psychotherapy for the treatment of anxiety disorders (3, 9). Yet, many patients completing individual or group CBT have residual symptoms and they, or their therapist, still feel that there is room for improvement (10). Old habits of thinking might resist CBT in those with high anxiety and limited or slow change in psychotherapy can occur even in the presence of a positive patient–therapist alliance and good adherence to treatment (11, 12). One of the factors involved in resistance to change could be that psychotherapists face the very difficult task of helping patients solve long standing emotional and irrational problems with treatment methods that are almost always based on rational and conscious communication.

### Preconscious Priming

People suffering from anxiety disorder have difficulties generating positive scenarios compared to control participants (13), which might account for part of the limited effectiveness of treatments where alternative cognitions or scenarios must be generated rationally by the patient, such as CBT. Priming is a technique by which a stimulus previously presented makes a related but new stimulus more readily available. This should be a potential candidate for helping participants generating or accessing different cognitions. This paradigm has been used mainly in cognitive research, particularly for word recognition. Typically, a word (e.g., cars) is presented, followed by either a word of the same semantic category (e.g., Ferrari) or a different semantic category (e.g., cattle). Results show that the word of the same semantic category is then recognized faster because it has been primed, i.e., is more readily accessible to consciousness (14). If priming can make words more readily available in laboratory word recognition tasks, it could logically produce similar effects in natural settings (15). Hence, the results of priming studies suggest that a repeated activation or priming of cognitions, attitudes, and related emotions can also render a set of positive cognitions and self-statements more readily available to patients.

Priming is postulated to be mostly a preconscious phenomenon (15). Research concerning preconscious processes mainly involves the effect of stimuli presented long enough to be perceived, but not long enough to enter consciousness. For example, Ohman and Soares (16) successfully induced phobic responses by presenting pictures of fearful faces under the threshold of conscious recognition. Masked priming (i.e., unconscious priming) is also more effective than non-masked priming at inducing affective effects (17).

### Alternative Preconscious Priming

The procedure presented in this study is termed APAP for Augmentation of Psychotherapy through Alternative Preconscious Priming. The APAP strategy is based on the notion that priming and masked priming phenomena could render a set of positive cognitions, attitudes, and emotions more available to patients undergoing CBT. A previous randomized controlled study (1) has shown that the addition of APAP to CBT facilitated cognitive change and was associated with increased positive cognitions and decreased negative cognition in social phobia. This study also suggested that patients with important residual anxiety following CBT could benefit more from APAP than patients with less residual anxiety. A recent case report also demonstrated the effectiveness of APAP in a patient with severe social phobia (18).

### Aims

The current case series adds to the previous randomized controlled trial in two ways: first, the subjects were treatment-resistant social anxiety disorder (SAD) or generalized anxiety disorder (GAD) patients still showing important residual symptoms and, second, a new computerized program available through the internet and linking all the steps of the procedure was employed. The following questions were explored: Were residual symptoms of SAD or GAD improved by the addition of APAP? Was the use of the APAP program available through internet viewed as user friendly? Was the possibility of individual work at home with the possibility of relying on a therapist through internet judged positively?

## Method

### Subjects

Nine SAD or GAD patients were recruited at a specialized anxiety clinic. This clinic receives patients deemed treatment resistant after two unsuccessful treatment attempts, either with drugs or psychotherapy, but more frequently following two unsuccessful pharmacological attempts. Patients were referred to the clinic by their physician and received individual or group CBT. The format, duration, and intensity of the treatment were personalized to the patient. Participants were referred to the study by their therapist (psychologist or psychiatrist) based on important residual symptoms.

Of the nine participants entering treatment, one participant desisted because of a lengthy stay outside of the county, leaving eight completers (age range: 22–71 years old; five women; four SAD, and four GAD). Diagnostics were obtained through clinical judgment of professionals and were confirmed through previous diagnostics.

Inclusion criteria were (1) being over the age of 18 years old; (2) having a primary diagnostic of SAD or GAD; (3) presenting residual symptoms after CBT despite good treatment adherence; and (4) no evidence of substance or alcohol abuse within the past 6 months. Participants were excluded if they had (1) a current primary mood disorder, except for premorbid dysthymia or (2) a history of psychotic disorder or eating disorder.

Participants gave consent prior to the beginning of the study and were assessed by the first and third author at baseline. Participants’ age and diagnostics are presented in Table 1. All names have been changed to preserve anonymity.

**Table 1:** **Participants’ age and diagnostics**.

Participants	Age	Diagnostics
Caroline	34	SAD
Natalia	22	GAD
Sharon	39	GAD, OCD, and SAD
Isabelle	31	SAD, dysthymia, and subclinical OCD
Geneviève	28	GAD, obsessional personality traits and dependant personality traits
Pierre	71	GAD, slight premorbid prefrontal perturbation
Claude	44	SAD
Haroun	32	SAD, dysthymia; ADD

Mean	37.63	–

*GAD, generalized anxiety disorder; SAD, social anxiety disorder; OCD, obsessive–compulsive disorder; ADD, attention-deficit disorder*.

### Measures

#### Beck Anxiety Inventory (BAI)

The BAI (19) is a 21-item questionnaire measuring the intensity of anxiety symptoms in the past week. As with the English version, the French version present a good internal consistency (α = 0.84) and a good test–retest fidelity (*r* = 0.63) (20).

#### Hamilton Anxiety Rating Scale (HARS)

The HARS (21) measures state anxiety using 14 items. These items cover the following dimensions: psychic anxiety, somatization, cognitive impairments, sleep difficulties, and depressive mood. The interrater reliability is excellent (*r* = 0.89) (21) and convergent validity with other measures of anxiety is good (*r* = 0.51). The French validation was used in this study (22).

#### Quality of Life Systematic Inventory (QLSI)

The QLSI (23) measures participant’s quality of life by measuring their capacity to reach their personal goals in nine different life spheres. The QLSI presents a good test–retest reliability (*r* = 0.84) and good divergent validity with social desirability (*r* between 0.11 and −0.13) (23).

#### Idiosyncratic Measure of Convictions in Thoughts

This measure was designed specifically for the study and was inspired by the postulate of cognitive theory according to which change in cognition brings change in emotions and behaviors (8). Many other studies have used this methodology (24, 25) and this type of measure has been previously validated (26). The idiosyncratic measure asks participants to name the thoughts that would be targeted by APAP and to rate the degree of conviction in each thought in percentage. Thoughts targeted by APAP were decided by the patient and the therapist (see Procedure), but the ratings were provided by the patients independently of therapist. The mean of the ratings was used as a total score to effectively compare participants.

### Procedure

The study was approved by the local ethics and research committees. Participants with residual symptoms still causing disturbances were approached at the end of their CBT and invited to participate in APAP. Participants were informed that APAP could help to free them from a cognitive rut by making different cognitions more available to them. If the participants agreed to participate, individual sessions with FB and the participant’s therapist were organized to explain the procedure. The participants received at least two sessions face to face explaining APAP, its rationale, and how the procedure could be adapted to their situation. Afterward, participants had the option of continuing the therapeutic work by themselves over the internet through the APAP website^1^ or by doing it partly or totally face to face with the therapist using the web interface together. Information was exchanged *via* the APAP website’s interface for patients choosing to work from the internet to help specify the targets of change. The therapeutic work proceeded as follows: (1) recap on difficulties regarding change in cognition and inner discourse and explanation of APAP rationale (this step occurred in person); (2) exploring the residual problematic cognitions and related emotions and mental imagery (case formulation with the patient and her/his therapist using the APAP website); (3) making a list of plausible alternative and desired personal cognitions (formulated by the patient with the input of her/his therapist using the APAP website); and (4) explaining the listening modalities, which were: passive, effortless listening, and at least 20–30 min a day for 8 weeks. The listening setting could be during a relaxing moment, for instance at bedtime, but could also be used before or during stressful situations.

The list of plausible alternatives created by the patient were mixed with relaxing music chosen by the patient. The music was 20–30 dB stronger than the plausible alternatives to actively mask it. Participants were given the option to change the music using the APAP website to prevent boredom and increase motivation. They were also required to hold a diary monitoring their adherence to the treatment. Participants were contacted weekly by the first author to verify their adherence and to respond to any queries. Questions concerning the website as well as information about life event of participants were collected during the weekly contacts and at end point (8 and 24 weeks). Also, 4–6 weeks after the beginning of the procedure, patients were evaluated and changes were made to the plausible alternatives, if judged necessary. The trial ended after 8 weeks and further follow-up was made at 24 weeks (16 weeks posttreatment).

## Results

### Change in Dysfunctional Thoughts and Anxiety Symptoms

Table 2 presents the mean degree of conviction in the different dysfunctional beliefs held by each APAP participants before the beginning of the procedure and at 24 weeks. Substantial decrease from pre to 16 weeks posttreatment in the mean conviction were observed and only one participant (Sharon) failed to improve in regard to cognitions, but remained stable. All participants showed an increase in quality of life as well as an overall decrease in anxiety symptoms (see Table 3). Decrease in anxiety symptoms was modest, with some participants greatly decreasing (e.g., Caroline on the BAI) and others showing increase in symptoms (e.g., Pierre on the HARS). No participants showed an increase on both the BAI and the HARS suggesting that there was at least some improvement with all participants. There was also no relapse during follow-up.

**Table 2:** **Conviction in dysfunctional thoughts pre-APAP and at 24 weeks**.

Participants	Triggers of anxiety	Dysfunctional thoughts	Conviction in dysfunctional thoughts pre-APAP (%)	Conviction in dysfunctional thoughts at 24 weeks (%)
Caroline	Talking in front of groups	I express myself badly; my voice sound weird; I feel overwhelmed	96.7	31.7

Natalia	Fear of failure, particularly in school	I have difficulties taking decisions; I have uncertainties about the future; I won’t be able	82.1	21.4

Sharon	Feeling judged when meeting people	I’m afraid people will be angry at me; I’m afraid people will say bad things about me; I’m afraid people are going to laugh at me	90	90

Isabelle	Cannot manage anxiety when starting a new job	I’ll always be anxious; I won’t adapt, I’ll fail again; others will think I’m incompetent	83.3	36.3

Geneviève	Anticipation about returning to work	I’m concerned about going back to work; I’ll be sick; I won’t be able to do anything	98.9	77.8

Pierre	Anxiety about parking	I won’t find a parking spot when I’ll get back; I won’t know where to park; my car will get vandalized	66	17

Claude	Feeling invaded in social situations	I feel invaded; I won’t control my life; I express myself badly	88.6	68.6

Haroun	Anticipation of anxiety during phone calls	I’m unable to express my objectives; I’m afraid of reaction; I’m afraid of making a phone call	64	44

Mean	–	–	83.7	48.4

**Table 3:** **Change in quality of life and anxiety symptoms during APAP**.

	QLSI		BAI			HARS		
	Baseline	24 weeks	Baseline	8 weeks	24 weeks	Baseline	8 weeks	24 weeks
Caroline	3	69	33	9	14	14	20	18
Natalia	3	71	14	11	5	17	11	7
Sharon	2	13	42	36	41	46	37	35
Isabelle	3	13	10	5	7	18	10	10
Geneviève	2	13	28	20	21	30	28	25
Pierre	2	48	35	22	20	14	24	25
Claude	3	13	9	16	20	30	28	21
Haroun	2	13	13	36	25	35	23	26

Mean	2.5	31.6	23	19.4	19.1	26	22.63	20.9

*QLSI, Quality of Life Systematic Inventory; BAI, Beck Anxiety Inventory; HARS, Hamilton Anxiety Rating Scale*.

### Patient’s Report and Their Perception of APAP and Its Website

Participants reported further gains that could be attributable to APAP. Sharon and Geneviève started doing some volunteer work. Isabelle returned to work while Sharon and Genevieve joined a return to work group after APAP. Claude decided to go back to work for the first time in 6 years and started a traineeship for people reintegrating the market place during the follow-up. Caroline made new friends and kept regular contact with them, as well as exposing her paintings. Finally, Haroun started reusing the phone.

There were also events not related to APAP that could have negatively impacted participants. Natalia moved, Pierre had some health problems, and his wife was hospitalized just before the end of the eighth week (he went on to have worries about health which were not targeted during his APAP trial) and Haroun was accused of sexual harassment.

Participants’ testimony revealed that they greatly appreciated the possibility of working autonomously with APAP while being able to rely on their therapist, if needed. They appreciated being listened to when they felt the need to change their therapeutic target and they underlined the importance of a fluid program that allows to make such changes according to the patient’s needs.

## Discussion

The present study sought to investigate the effectiveness of an augmentation of psychotherapy through alternative preconscious priming (APAP) intervention following CBT in treatment-resistant patients with residual symptoms. The procedure was supported by a patient–clinician collaborative website specifically designed for this purpose. Results showed improvement in targeted cognitions, quality of life, and anxiety symptoms during APAP that were maintained at follow-up, indicating that APAP could be used to help diminish residual symptoms of SAD and GAD following CBT. The users also positively appraised the procedure and the website. This study suggests that APAP could be a useful addition to CBT, particularly for those affected by resistant problematic cognition and internal discourse. Conceptually, APAP rationale is similar to CBT as it aims to change problematic cognitions postulated to be related to negative emotion and behaviors. To our knowledge, no other study has been reported using a rationale of preconscious priming or using repeated preconscious listening in the context of psychotherapy augmentation.

As previously mentioned by Tulving and Schacter (15), priming can have practical therapeutic uses. Change in cognitions targeted by APAP suggests sound internal validity of the intervention, APAP being the only intervention received by participants which targeted these thoughts. The results replicate the change in cognition found in a previous validation of APAP (1). While the reduction in anxiety was modest, the improvement in quality of life was substantial for some participants and gains in functioning were achieved by all participants and maintained at follow-up. This suggest that 8 weeks of passive repeated preconscious listening could have lasting effects beyond the use of a simple listening period. Results also suggest that comorbidities (particularly OCD and personality traits/disorder) or life events could diminish the effectiveness of APAP, as they do with any therapy. Yet even those with comorbidities or encountering adverse life events improved in some way.

Like APAP, other internet based treatments have also shown effectiveness (27), even compared with the usual treatments (28). These results are encouraging for internet intervention and suggest that some people might respond better when the setting is different then traditional psychotherapy.

Preconscious processes are also implicated in other anxiety disorders. People with obsessive–compulsive disorder show preconscious learning difficulties using a procedural task compared to healthy controls.^2^ Change in preconscious processes (in this case, attention processes) can effectively reduce the risk of developing subsequent posttraumatic stress disorder (29). This tends to show the importance of preconscious processes in anxiety disorders as an area to investigate, particularly for those who are treatment resistant.

### Limits

In this case series, it is hard to distinguish the gains from APAP from the influence of other factors, for example, the residual effect of the participants’ previous CBT. However, APAP has been previously validated as an effective procedure beyond residual effect of CBT and bogus priming (1). The small sample size and the nature of the study prevent us from generalizing the observation reported. A future replication should thus use a larger sample. The reliance of self-report assessment prevents us from distinguishing theses results from participants’ biases, since they were not blind to the study hypothesis. Future studies should address this issue.

### Strengths and Clinical Implications

This is the first time APAP has been used to treat GAD, the previous study focusing only on SAD (1), implying that APAP can be used to target various anxiety symptoms. This was also the first time that APAP was delivered over the internet, which was appreciated by the participants and allowed flexibility in the program. The previous utilization of APAP necessitated the use of a recording studio, limiting the capacity to adapt APAP to individual needs over time.

### Future Recommendations

Future studies could include randomized controlled trial of APAP with a larger sample size, which would also aim to understand which clinical characteristic predicts meaningful response to APAP. Other psychiatry disorders presenting problematic internal discourse could potentially benefit from a similar procedure. APAP could also be applied in other formats (e.g., as a self-help procedure or combined with medication).

## Conclusion

Alternative Preconscious Priming could be a new and useful strategy to facilitate cognitive change and augment the effects of psychotherapy. In the present study, it helped to diminish participants’ anxiety and improved their quality of life. Patients presenting comorbid OCD and personality disorder might not benefit from APAP as much as other patients. The use of a website was judged appropriate to facilitate access and autonomy, but improvements are still needed to increase program friendliness. Further studies, especially controlled studies, are warranted to assess APAP usefulness and its possible applications.

## Ethics Statement

The study was approved by the comité d’éthique de la recherche de l’institut universitaire en santé mentale de Montréal. This ethics committee has been dissolved due to recent changes in the administrative structure. It is now called: comité d’éthique de la recherche du centre intégré universitaire de santé et des services sociaux de l’est-de-Montréal. Participants were approached at the end of their psychotherapy and they were invited to participate in the current study. They were given the consent form and were told to read it thoroughly before agreeing. The research team answered any question concerning the consent form, and all was done to ensure that participants understood that they were free to disengage from the study at any points and that there would be no consequences to their disengagement.

## Author Contributions

MZ, FB, FA, and YK contributed to the design of the work. MZ and FB collected the data. MZ analyzed the data. MZ, J-SA, FB, FA, KO, and YK participated in the interpretation of the data. J-SA drafted the manuscript. MZ, FB, FA, KO, and YK revised the manuscript critically. All the authors approved the final version of the manuscript.

## Conflict of Interest Statement

The authors declare that the research was conducted in the absence of any commercial or financial relationships that could be construed as a potential conflict of interest.

## Footnotes

^1^The program is available in French freely to psychotherapist upon request to the corresponding author.

^2^Dulude G, Bédard M-A, O’Connor K, Audet J-S. Over facilitation of unadapted cognitive processes in obsessive compulsive disorder as assessed with the computerized mirror pointing task. *J Psychiatr Res* (In revision).
